# Prevalence and seasonality of viral respiratory infections in a temperate climate region: A 24‐year study (1997–2020)

**DOI:** 10.1111/irv.12972

**Published:** 2022-02-16

**Authors:** Laura García‐Arroyo, Núria Prim, Marga Del Cuerpo, Pilar Marín, Maria Carme Roig, Mnontserrat Esteban, Rosa Labeaga, Neus Martí, Carla Berengua, Ignasi Gich, Ferran Navarro, Núria Rabella

**Affiliations:** ^1^ Microbiology Department. Hospital de la Santa Creu i Sant Pau, Universitat Autònoma de Barcelona (UAB) Sant Pau Biomedical Research Institute (IIB Sant Pau) Barcelona Spain; ^2^ CIBER Epidemiología y Salud Pública (CIBERESP), Clinical Epidemiology and Public Health Department, Hospital de la Santa Creu i Sant Pau Sant Pau Biomedical Research Institute (IIB Sant Pau) Barcelona Spain; ^3^ Departament de Genètica i Microbiologia Universitat Autònoma de Barcelona (UAB) Barcelona Spain

**Keywords:** climate, epidemiology, influenza, prevalence, respiratory viruses, seasonality

## Abstract

**Background:**

Few long‐term reports have been published on the epidemiology of respiratory viruses despite their frequent involvement in extremely common infections. The aim here was to determine the frequency and distribution of respiratory viruses in a temperate climate area (Barcelona, Spain) throughout a 24‐year period.

**Methods:**

We collected data on all respiratory viruses detected from 1997 to 2020 in our institution. Clinical specimens were analyzed mainly by conventional techniques, and molecular techniques were also used.

**Results:**

Of the 59,579 specimens analyzed, 21,382 (35.9%) were positive for at least one virus. The number of positive samples during cold months was significantly higher than in warm months. Respiratory virus infections were detected in patients of all ages, above all in children under 3 years of age, who were most frequently infected with the respiratory syncytial virus, whereas Influenza A virus predominated in the other groups, especially in adults. A clear demographic and seasonal pattern was established for some viruses. Circulation of other respiratory viruses during the FLUAV H1N1pdm09 and SARS‐CoV‐2 pandemics was observed.

**Conclusions:**

This long‐term study provides new knowledge about the prevalence of respiratory viruses in a Mediterranean region. Throughout the study period, the frequency of some viruses remained constant, whereas others varied with the year. A clear demographic and seasonal pattern was established for some viruses. Patients suffering from severe respiratory infections should be examined for a range of respiratory viruses regardless of gender, age, or season.

## 1 INTRODUCTION

Respiratory viral infections are extremely common and among the main causes of medical consultations, especially in pediatric patients. They have a wide range of clinical presentations, ranging from common colds to severe lower respiratory tract infections. As the same clinical presentation may be caused by different viruses and the same virus may manifest with different symptoms, microbiological diagnosis is essential to determine the etiological agent causing the infection.^1^ The most common respiratory viruses are respiratory syncytial virus (RSV), adenoviruses (AdV), influenza virus types A and B (FLUAV and FLUBV), human metapneumovirus (hMPV), parainfluenza virus types 1–3 (PIV‐1, PIV‐2, and PIV‐3), rhinoviruses (RV), enteroviruses (EV), and coronaviruses (CoV). Classical virological diagnosis of respiratory infections is based on cell culture and/or antigen detection techniques. Molecular techniques have improved the capacity to detect these and other viruses in respiratory tract specimens, especially those that cannot be identified by conventional methods.^2^, ^3^

The few long‐term studies on respiratory virus epidemiology published to date^4^, ^5^, ^6^, ^7^, ^8^, ^9^ show seasonal patterns of circulation in defined geographical areas. However, most reports cover shorter periods.^10^, ^11^, ^12^, ^13^, ^14^ The epidemiological studies carried out in Spain are restricted to a few seasons,^15^ certain viruses,^16^ or particular groups of patients,^17^ and to our knowledge, none have focused on the prevalence of several respiratory viruses within a broad population over a long period of time.

The aim of the present study was to determine the frequency and distribution of respiratory viruses in a Mediterranean area throughout a 24‐year period, including the first months of the SARS‐CoV‐2 pandemic.

## 2 MATERIALS AND METHODS

### 2.1 Study design and data collection

We collected all the available data on respiratory viruses detected from January 1997 to March 2020 in our institution, a tertiary referral teaching hospital covering an area of 407,550 inhabitants in Barcelona (Spain). We analyzed the circulation of respiratory viruses, including frequency and seasonal distribution, the relationship between the different viruses detected, and two baseline parameters of patients (age and sex). The Ethics Committee of *Hospital de la Santa Creu i Sant Pau* approved the research (approval number: IIBSP‐VIR‐2014‐41) and waived the need for consent.

Patients were grouped according to their age: groups A (under 6 months), B (6–12 months), C (1–2 years), D (3–5 years), E (6–17 years), F (18–29 years), G (30–39 years), H (40–59 years), and I (over 60 years). All patients under 18 years of age were assigned to the pediatric population. Patients were classified as of unknown age when this information was unavailable.

The clinical specimens analyzed were nasopharyngeal aspirates, nasal and pharyngeal exudates, bronchoalveolar lavages, and lung biopsies. The conventional techniques routinely used to identify respiratory viruses were immunochromatography, immunofluorescence (IF), and cell culture (CC), as described in the literature.^18^ The respiratory viruses detected using these techniques were RSV, AdV, FLUAV, FLUBV, PIV1, PIV2, PIV3, RV, and EV; screening for hMPV was included from December 2008. At the start of 2009, an in‐house PCR was introduced to detect FLUAV, FLUAV H1N1pdm09, and FLUBV. Since February 2010, the molecular detection of these viruses has been performed by GeneXpert® Dx (Cepheid), and since January 2015, this kit has also targeted RSV. A panel for the molecular detection of respiratory viruses (FilmArray™) was introduced in the routine workflow of the laboratory in May 2014, allowing the detection of PIV4 and different coronaviruses (HuCoV‐229E, HuCoV‐HKU1, HuCoV‐NL63, and HuCoV‐OC43). At the beginning of 2020, several molecular methods were set up for the detection of SARS‐CoV‐2 (GeneXpert®, Simplexa®, VIASURE® Real Time PCR Detection Kit, SARS‐CoV‐2 ELITe MGB® Kit, cobas® SARS‐CoV‐2 Test, and Alinity m SARS‐CoV‐2 assay).

Specimens received from March 2020 were only studied for the presence of SARS‐CoV‐2 due to the exceptionality of that year, and they were analyzed separately.

### 2.2 Statistical analyses

Statistical analysis was performed by means of SPSS v26 (IBM Corp, Armonk, New York, USA). Continuous data and categorical data were analyzed using the *t* test and Chi‐square test, respectively.

## 3 RESULTS

### 3.1 General results

In the 1997–2019 period, 59,579 respiratory specimens were received by the laboratory, with a median of 2,590 per year (range 952–4,883). Of these, 49,712 samples were processed by conventional methods and the other 9,867 only by molecular methods. A total of 21,382 samples (35.9%) were positive for at least one virus. Overall, 21,939 virus detections were made, with more than one identified in 551 specimens (2.6%). The rate of positivity was significantly higher in samples acquired during the cold versus warm months, matching the seasonal distribution of samples received (*p* < 0.001).

In 2020, a total of 66,616 respiratory specimens were collected, 2,903 during the three first months of the year, and 832 (28.6%) respiratory virus detections were made. All specimens received after the WHO declaration of the SARS‐CoV‐2 pandemic were analyzed only for SARS‐CoV‐2. A total of 6,566 samples were positive for SARS‐CoV‐2 in 2020.

### 3.2 Seasonal distribution of respiratory viruses

The different respiratory viruses identified throughout the study period are shown in Table 1. The most frequently detected were FLUAV (31.4%) and RSV (28.7%), which were co‐detected in 25.5% (118/462) of all the samples that tested positive for more than one virus.

**TABLE 1:** Yearly distribution of the respiratory viruses detected throughout the period under study (n = 21 939)

Viruses^a^	1997	1998	1999	2000	2001	2002	2003	2004	2005	2006	2007	2008	2009	2010	2011	2012	2013	2014	2015	2016	2017	2018	2019	Total
RSV, n (%)	165	362	178	313	201	265	230	231	315	266	294	284	239	208	233	115	109	196	276	413	413	389	465	6,160
	(47.1)	(36.5)	(17.1)	(29)	(38.7)	(41.6)	(32.6)	(46.8)	(38.2)	(35.9)	(30.6)	(39)	(15.5)	(28.3)	(25.3)	(16.3)	(17.3)	(25.2)	(24.8)	(26.9)	(30.2)	(21.2)	(27.7)	(28.1)
FLUAV, n (%)	22	381	538	610	42	89	216	20	182	112	226	81	728	130	138	308	89	406	441	601	497	640	870	7,367
	(6.3)	(38.4)	(51.7)	(56.4)	(8.1)	(14)	(30.6)	(4)	(22.1)	(15.1)	(23.5)	(11.1)	(47.2)	(17.7)	(15)	(43.6)	(14.1)	(52.3)	(39.7)	(39.1)	(36.3)	(34.9)	(51.8)	(33.6)
FLUBV, n (%)	25	11	23	1	9	71	3	0	41	44	58	86	101	2	176	52	208	4	110	270	156	518	37	2006
	(7.1)	(1.1)	(2.2)	(0.1)	(1.7)	(11.1)	(0.4)		(5)	(5.9)	(6)	(11.8)	(6.5)	(0.3)	(19.1)	(7.4)	(33.1)	(0.5)	(9.9)	(17.6)	(11.4)	(28.3)	(2.2)	(9.2)
AdV, n (%)	58	123	152	68	101	79	122	67	99	133	199	126	218	132	146	83	91	44	84	66	112	101	120	2,524
	(16.6)	(12.4)	(14.6)	(6.3)	(19.5)	(12.4)	(17.3)	(13.6)	(12)	(17.9)	(20.7)	(17.3)	(14.1)	(18)	(15.8)	(11.8)	(14.5)	(5.7)	(7.6)	(4.3)	(8.2)	(5.5)	(7.1)	(11.5)
EV, n (%)	55	78	102	66	104	81	71	51	88	84	66	81	81	120	72	46	27	29	36	38	55	29	27	1487
	(15.7)	(7.9)	(9.8)	(6.1)	(20)	(12.7)	(10.1)	(10.3)	(10.7)	(11.3)	(6.9)	(11.1)	(5.2)	(16.3)	(7.8)	(6.5)	(4.3)	(3.7)	(3.2)	(2.5)	(4)	(1.6)	(1.6)	(6.8)
RV, n (%)	0	1	4	0	4	37	33	62	56	47	71	30	65	55	46	54	37	57	73	78	57	65	75	1006
		(0.1)	(0.4)		(0.8)	(5.8)	(4.7)	(12.6)	(6.8)	(6.3)	(7.4)	(4.1)	(4.2)	(7.5)	(5)	(7.6)	(5.9)	(7.3)	(6.6)	(5.1)	(4.2)	(3.5)	(4.5)	(4.6)
PIV1, n (%)	1	6	13	7	34	6	13	28	13	2	6	1	8	1	3	0	13	0	7	0	2	1	9	174
	(0.3)	(0.6)	(1.2)	(0.6)	(6.6)	(0.9)	(1.8)	(5.7)	(1.6)	(0.3)	(0.6)	(0.1)	(0.5)	(0.1)	(0.3)		(2.1)		(0.6)		(0.1)	(0.1)	(0.5)	(0.8)
PIV2, n (%)	16	7	17	1	5	1	4	0	9	0	15	3	15	1	2	2	2	1	6	5	3	8	4	127
	(4.6)	(0.7)	(1.6)	(0.1)	(1)	(0.2)	(0.6)		(1.1)		(1.6)	(0.4)	(1)	(0.1)	(0.2)	(0.3)	(0.3)	(0.1)	(0.5)	(0.3)	(0.2)	(0.4)	(0.2)	(0.6)
PIV3, n (%)	8	22	14	15	19	8	14	35	21	53	26	32	28	33	33	21	31	13	35	23	27	26	16	553
	(2.3)	(2.2)	(1.3)	(1.4)	(3.7)	(1.3)	(2)	(7.1)	(2.5)	(7.2)	(2.7)	(4.4)	(1.8)	(4.5)	(3.6)	(3)	(4.9)	(1.7)	(3.1)	(1.5)	(2.0)	(1.4)	(1)	(2.5)
hMPV, n (%)	‐	‐	‐	‐	‐	‐	‐	‐	‐	‐	‐	5	60	53	73	25	22	27	44	42	46	56	58	511
												(0.7)	(3.9)	(7.2)	(7.9)	(3.5)	(3.5)	(3.5)	(4)	(2.7)	(3.4)	(3.1)	(3.5)	(2.3)
Total of viruses	350	991	1,041	1,081	519	637	706	494	824	741	961	729	1543	735	922	706	629	777	,112	1,536	1,368	1,833	1,681	21,915^b^
Samples, n	952	1,943	1,825	2,045	1,930	1,936	2,265	1,745	2,315	2,077	2,438	2,131	4,152	2,246	2,609	2,047	2,088	2,345	3,069	4,224	3,788	4,883	4,526	59,579

^a^ The percentage of each virus is calculated considering the number of total viruses each year.

^b^ The total number does not include five PIV4 and 19 non‐typed PIV.

RSV, FLUAV, AdV, EV, and PIV3 were detected every year throughout the period prior to the SARS‐CoV‐2 pandemic. RSV constituted 15.5% to 47.1% of all detected respiratory viruses per year, the detection rate being significantly higher in 1997 and 2004 (*p* < 0.001); FLUAV ranged from 4% to 56.4%, with rates highest in 1999, 2000, 2014, and 2019 (*p* < 0.001); AdV, 4.3% to 20.7%, with a peak in 2007 (*p* < 0.001); EV, 1.6 to 20%, being highest in 2001 and 2010 (*p* < 0.001); and PIV3, 1% to 7.2%, with the highest rate in 2004 and 2006 (*p* < 0.001). After the introduction of routine testing for hMPV, this virus was detected every year (2008–2019) at rates ranging from 2.7% to 7.9%, with the highest in 2010 and 2011 (*p* < 0001) (Table 1).

The other respiratory viruses were not detected every year. FLUBV was not observed in 2004 but otherwise constituted 0.1% to 33.1% of all viruses detected each year, the highest detection rates being in 2013, 2016, and 2018 (*p* < 0.001); RV ranged from 0.1% to 12.6%, with the highest rate observed in 2004 (*p* < 0.001); PIV‐1, 0.1% to 6.6%, with peaks in 2001 and 2004; and PIV‐2, 0.1% to 4.6%, peaking in 1997 (*p* < 0.001) (Table 1).

**FIGURE 1:**
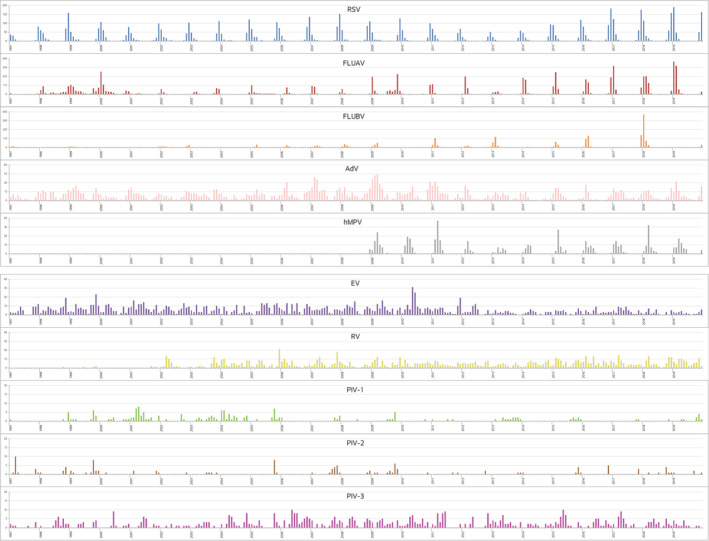
Respiratory viruses during the 23‐year period study (1997/2019). Monthly distribution of each respiratory virus detected (part 1 of 2). The depicted area for each virus is adapted to the total number of viruses detected. The large line on each year marks every January

**FIGURE 2:**
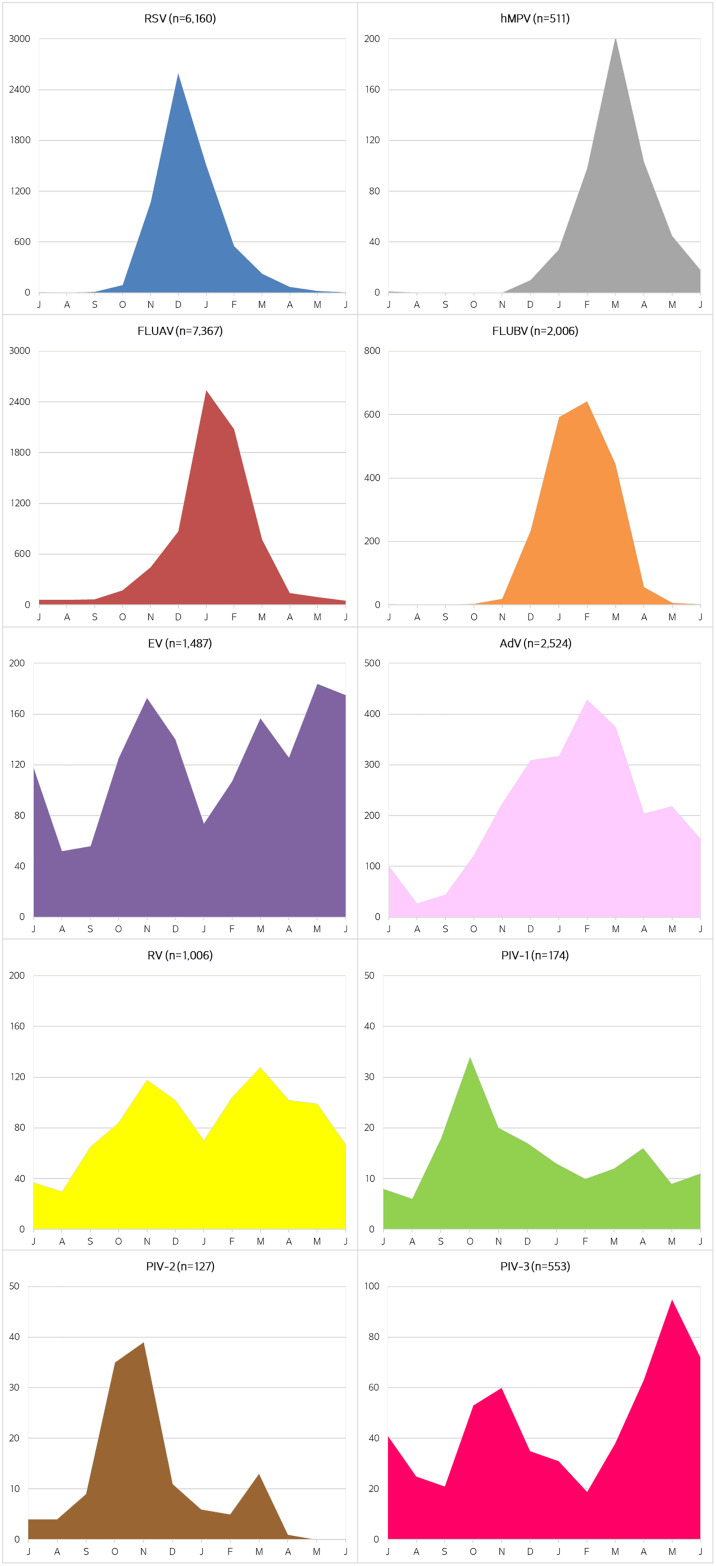
Seasonal pattern of each respiratory virus (shown from July to June). The depicted area for each virus is adapted to the total number of viruses detected

The monthly distribution of each respiratory virus and their seasonal patterns during the 23 years prior to the SARS‐CoV‐2 pandemic are shown in Figures 1 and 2, respectively. The relation between season and prevalence was statistically significant for all viruses (*p* < 0.001). Among the viruses detected in the cold months, RSV detection was highest from November to January, reaching a maximum in December. The prevalence of FLUAV was highest from December to March, normally reaching a peak in January, whereas for FLUBV, it was from January to March, with a peak usually 1 month after FLUAV. hMPV was most prevalent from February to May, and PIV‐2, from September to December, with a peak usually in November.

The other respiratory viruses in this study were detected throughout the year: AdV was most prevalent from November to May; EV and RV usually peaked in autumn and spring; PIV‐1, from September to April with a peak in October; and PIV‐3, from April to June and October to December.

### 3.3 Respiratory viruses during the FLUAV H1N1 pdm09 pandemic

The circulation of other respiratory viruses during the FLUAV H1N1 pdm09 pandemic (April 2009 to March 2010) was analyzed. Overall, 3,512 samples were screened for respiratory viruses during this period, 1,068 of which tested positive (30.4%) with a total of 1,080 virus detections (Table 2). Four‐hundred seven specimens tested positive for FLUAV by PCR. IF/CC resulted in 673 virus detections, RSV being the most frequently detected, followed by AdV and EV. During the pandemic, FLUAV was the predominant respiratory virus detected (43%), reaching a peak in November 2009, whereas RSV, AdV, EV, RV, hMPV, and PIV constituted 57% of the detections (Table 2), with the expected seasonal distribution.

**TABLE 2:** Respiratory viruses detected during the FLUAV H1N1 pandemics (April 2009–march 2010)

Samples/detection method		Conventional, *n*	Molecular, *n*	Total
Total		1,961	1,551	3,512
Positive		661	407	1,068
Viruses, *n* (%)^b^	RSV	260	ND	260
	FLUAV	57	407	464^a^
	FLUBV	2	0	2
	AdV	128	ND	128
	EV	79	ND	79
	RV	54	ND	54
	PIV‐1	8	ND	8
	PIV‐2	13	ND	13
	PIV‐3	26	ND	26
	hMPV	46	ND	46
Total of viruses, *n*		673	407	1,080^b^

Abbreviation: ND, not detected by the molecular technique used.

^a^ FLUAV corresponded to 43% of the total of viruses detected in this period.

^b^ Two viruses were detected in 12 samples.

### 3.4 Correlation between respiratory viruses and the age/sex of patients

The highest number of samples was collected from patients over 60 years (group I) and the lowest from those between 18–29 years (group F). The detection frequency for each respiratory virus varied between the different age groups (Table 3), the differences being statistically significant for all studied viruses (*p* < 0.001). Maximum virus detection occurred in patients of groups B and C (i.e., infants from 6 months to 2 years), with 51.7% and 51.3% of samples testing positive, respectively. At least one virus was detected in 38.9%, 44.8%, and 32.1% of the samples from groups A, D, and E, respectively. RSV was the most frequently detected virus in patients under 3 years of age. FLUAV was predominant in the other groups, especially in patients over 18 years of age.

**TABLE 3:** Distribution of viruses considering the range of age. Samples from patients of unknown age (n = 492) were not included

Virus/age group		Group A < 6 m	Group B 6–12 m	Group C 1–2 y	Group D 3–5 y	Group E 6–17 y	Group F 18–29 y	Group G 30–39 y	Group H 40–59 y	Group *I* > 60 y	Total
Specimen, *n*		13,874	5,219	8,162	2,872	3,918	1,675	2,514	6,216	14,637	59,087
Positive specimen, *n* (%)^a^		5,398	2,699	4,185	1,288	1,259	442	648	1,445	3,821	21,185
		38.9%	51.7%	51.3%	44.8%	32.1%	26.4%	25.8%	23.2%	26.1%	
Viruses, *n* (%)^b^	RSV	2,625	1,001	1,137	183	77	39	71	191	762	6,086
		47.3%	35.6%	26.4%	13.9%	6.0%	8.7%	10.9%	13.0%	19.7%	
	FLUAV	921	543	1119	471	466	296	401	871	2225	7,313
		16.6%	19.3%	25.9%	35.9%	36.3%	66.2%	61.4%	59.5%	57.4%	
	FLUBV	117	72	182	202	351	54	105	225	695	2,003
		2.1%	2.6%	4.2%	15.4%	27.3%	12.1%	16.1%	15.4%	17.9%	
	AdV	405	617	998	211	158	14	21	34	33	2,491
		7.3%	22.0%	23.1%	16.1%	12.3%	3.1%	3.2%	2.3%	0.9%	
	EV	605	217	359	106	95	6	9	28	36	1,461
		10.9%	7.7%	8.3%	8.1%	7.4%	1.3%	1.4%	1.9%	0.9%	
	RV	402	122	182	57	72	16	24	53	75	1,003
		7.2%	4.3%	4.2%	4.3%	5.6%	3.6%	3.7%	3.6%	1.9%	
	PIV‐1	69	21	32	14	9	2	3	10	5	165
		1.2%	0.7%	0.7%	1.1%	0.7%	0.4%	0.5%	0.7%	0.1%	
	PIV‐2	44	17	26	18	9	0	1	6	6	127
		0.8%	0.6%	0.6%	1.4%	0.7%	‐	0.2%	0,4%	0.2%	
	PIV‐3	204	98	117	14	28	18	15	31	23	548
		3.7%	3.5%	2.7%	1.1%	2.2%	4.0%	2.3%	2.1%	0.6%	
	hMPV	157	101	161	37	20	2	3	15	15	511
		2.8%	3.6%	3.7%	2.8%	1.6%	0.4%	0.5%	1.0%	0.4%	
Mixed detection		154	112	135	28	30	5	5	19	54	542
Total of viruses, *n*		5,549	2,809	4,313	1,313	1,285	447	653	1,464	3,875	21,708^c^

Abbreviations: m, months; y, years.

^a^ Percentage of positive samples considering each group of age.

^b^ Percentage of each virus out of the total of viruses in each group of age.

^c^ The total number does not include five PIV4 and 19 non‐typed PIV.

**FIGURE 3:**
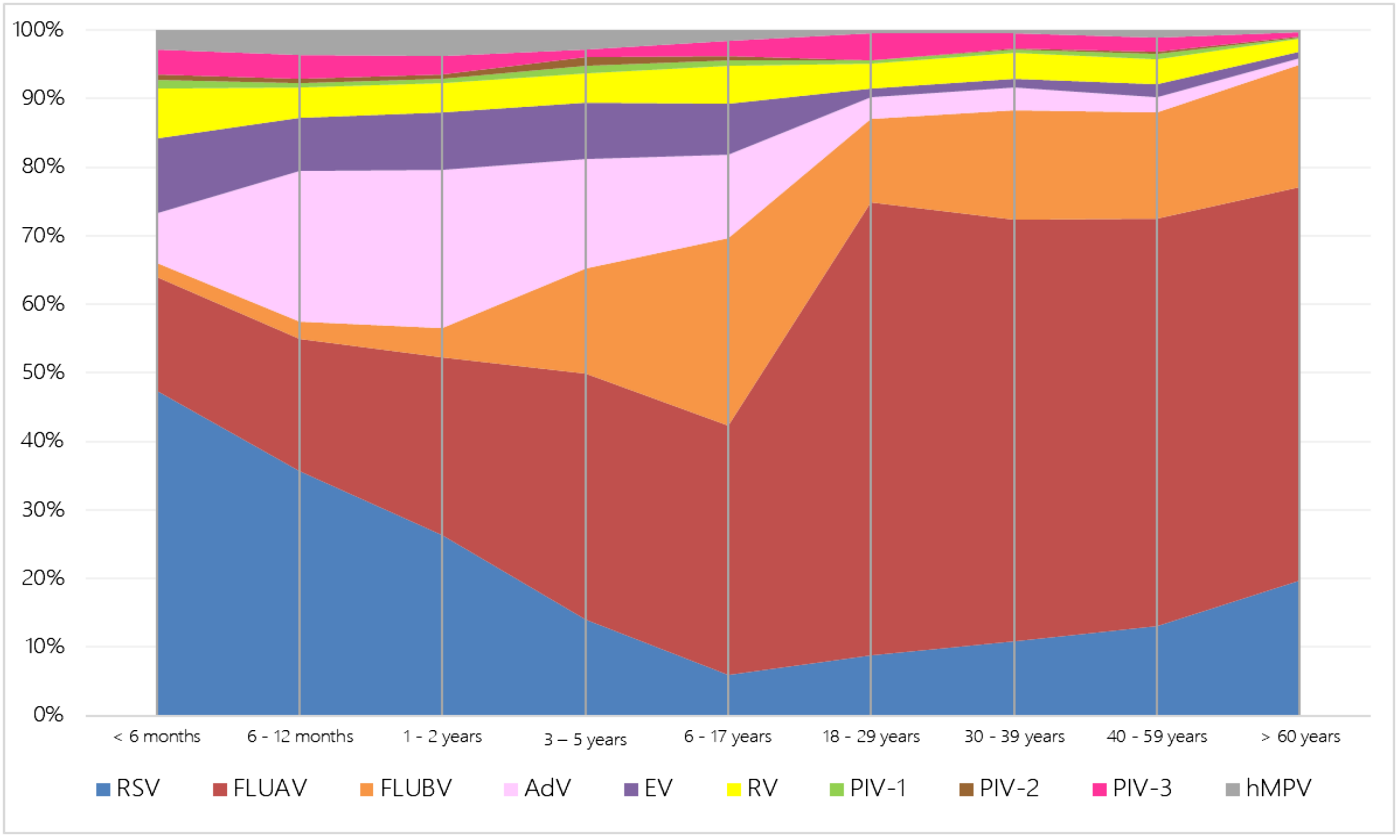
Occurrence of each respiratory virus in relation to the group of age. Respiratory viruses are distributed in percentage according to the age

The distribution of each virus was analyzed within the different age groups (Figure 3). RSV was the most frequent virus in infants up to 6 months of age (group A) (*p* < 0.001), with 43.1% (2,625/6,086) of all RSV cases identified in this group. The frequency of hMPV was highest in infants of group C (31.5%; 161/511) and group A (30.7%; 157/511) (*p* < 0.001). Of all FLUAV cases, 30.4% (2,225/7,313) and 15.3% (1,119/7,313) corresponded to groups I and C, respectively, and 34.7% of FLUBV detections (695/2,003) to group I. AdV was detected most frequently in group C (*p* < 0.001), with 40.1% (998/2,491) of all positive samples corresponding to this group of infants. Detection of EV and RV was higher in pediatric patients than in adults (*p* < 0.001). Both EV (*n* = 1461) and RV (*n* = 1003) were most frequently detected in group A (detection rates of 41.4% and 40.1%, respectively). Detection of parainfluenza viruses was also higher in pediatric patients than in adults (*p* < 0.001), with the highest rates obtained in group A: 41.8% PIV‐1 (69/165), 34.6% PIV‐2 (44/127), and 37.2% PIV‐3 (204/548).

Regarding the gender of patients, 54.6% (*n* = 32,587) and 44.8% (*n* = 26,688) of specimens were from males and females, respectively. Respiratory viruses were detected in 11,394 samples from males and 9,873 from females. Although more specimens were received from male patients, the percentage of positivity was higher for females (36.9%) than for males (34.9%) (*p* < 0.001). Differences in the positivity rates between the genders stemmed from the groups of patients more than 18 years old.

### 3.5 Circulation of other respiratory viruses during the SARS‐CoV‐2 pandemic

The circulation of other respiratory viruses besides SARS‐CoV‐2 was recorded in 2020. This year was analyzed separately and divided in two periods, before and after the WHO declaration of the SARS‐CoV‐2 pandemic on March 15, 2020. In the first period, 2,903 specimens were analyzed for the presence of respiratory viruses other than SARS‐CoV‐2, and 832 (28.6%) of them were positive: FLUAV (*n* = 401), FLUBV (*n* = 223), RSV (*n* = 104), AdV (*n* = 46), hMPV (*n* = 28), RV (*n* = 16), EV (*n* = 13), and PIV‐1 (*n* = 1). In the second period, 905 specimens were analyzed for the presence of respiratory viruses other than SARS‐CoV‐2, and 30 (3.3%) of them were positive: FLUAV (*n* = 9), AdV (*n* = 8), RV (*n* = 7), FLUBV (*n* = 3), hMPV (*n* = 1), EV (*n* = 1), and PIV‐1 (*n* = 1). The results from 2020 were not included in the statistical analysis.

## 4 DISCUSSION

The aim of the present study was to describe the frequency and distribution of respiratory viruses in a specific geographical area with a temperate climate over a 24‐year period up to 2020. In the 23 years prior to the SARS‐CoV‐2 pandemic, the overall positivity rate was 36.8% of all the samples analyzed, which is in accordance with other studies also using conventional methods.^4^, ^5^, ^10^, ^11^, ^12^, ^19^ The main differences with other reports are due to the different viruses evaluated, as not all include RV, EV, or herpes viruses.^4^, ^5^, ^11^ As expected, our positivity rates are lower than in studies using molecular techniques, which are more sensitive and can identify a wider range of viruses than conventional methods.^6^, ^13^, ^14^, ^20^, ^21^, ^22^

The phenomenon of respiratory viral interference, in which infection by a particular virus may interfere with the timing or rate of other viral outbreaks, is well documented.^22^ The long period of our study allowed us to observe variations in frequency and distribution of respiratory viruses over time. The highest positivity rates for some viruses were observed in years with a low circulation of FLUAV; this was the case for PIV‐1 in 2001 and 2004, AdV and EV in 2001, and RV and PIV‐3 in 2004. Van Asten et al^23^ reported that when FLUAV infections appeared early, RSV outbreaks tended to be delayed. In contrast, in our experience, the seasonal pattern of RSV was similar every year, regardless of FLUAV.

The detection frequency of the viruses in our study is similar to that of previous reports.^5^, ^9^, ^24^ The differences observed may be explained by the type of population under study, the season in which the study was performed, its length, and/or the methodologies used.^4^, ^10^, ^12^, ^19^ Thus, we observed a lower RSV frequency compared to studies focusing only on a pediatric population.^4^, ^9^, ^10^, ^12^ The detection frequency of FLUAV and FLUBV also differed from the literature, as although infections caused by these viruses follow a yearly epidemical pattern, their intensity fluctuates.^4^, ^6^, ^7^, ^9^, ^12^ Furthermore, some viruses were found in lower frequencies (especially hMPV, RV, EV, and some PIV) than in other studies using molecular methods.^13^, ^20^, ^21^, ^22^

In temperate climates, respiratory viruses are usually expected during the cold months, whereas in tropical climates, they may be found throughout the year or are associated with the rainy season.^4^, ^7^, ^9^, ^12^, ^19^, ^25^, ^26^ In the present study, RSV, influenza viruses, and hMPV were mainly detected in winter and early spring and hardly detected in summer, in agreement with other studies performed in temperate climates.^6^, ^12^, ^22^, ^27^, ^28^, ^29^ RSV was the first virus detected at the start of the cold season, displaying a marked seasonal distribution between November and January. FLUAV was found mainly between December and March. Although not detected every year, FLUBV circulated mainly between January and March, usually after the FLUAV epidemics,^30^ and was followed by hMPV, usually between February and May.

Infections caused by other respiratory viruses such as AdV and PIV‐3 were observed throughout the year, in accordance with other studies in temperate climates.^3^, ^6^, ^28^ EV and RV showed a bimodal distribution, with seasonal peaks in autumn and spring, as previously reported.^3^, ^6^ Other PIVs do not regularly circulate throughout the year or every year and vary between different geographic areas.^10^, ^28^ In our study, PIV‐1 was detected essentially in autumn and nearly every year, whereas PIV‐2 was mainly found between September and December.

In April 2009, the WHO announced the outbreak of the FLUAV H1N1 pdm09 pandemic, which lasted until March 2010. During this period, when FLUAV detection was a priority, it was the most frequently detected virus (43% of all respiratory viruses). However, over half of the respiratory viruses detected during the 2009/2010 pandemic corresponded to viruses other than FLUAV, in agreement with other regional laboratories.^31^ Given that the clinical manifestations of influenza viruses are not specific^21^ and the possibility of simultaneous circulation, screening for other respiratory viruses should also be carried out during epidemics/pandemics.

Overall, the highest positivity rates were observed in the pediatric population. All respiratory viruses may cause mild and self‐limited reinfections throughout a lifespan without requiring medical consultation, and therefore, the majority remains undetected. RSV was mainly diagnosed in children under 3 years of age, which may be partially explained by the high percentage of pediatric specimens (71.5%) included in our study, but also reflects that RSV infections are the main cause of respiratory infection (bronchiolitis) in infants.^10^ An increase in active screening of patients of all ages for RSV has brought to light its role in respiratory infections in adults.^5^, ^8^

We observed an increasing tendency in FLUAV detection with patient age, as described in the literature.^5^, ^22^, ^29^ This may be explained by the fact that new variants of influenza viruses appear each year and previously developed immunization may not be effective against the new variants.

Our results showed that children aged 5 to 18 years were the population with most FLUBV infections, which is in agreement with other studies.^10^, ^32^ Infections caused by AdV or hMPV were diagnosed mainly in children under 3 years. RV and EV infections were also predominantly detected in pediatric populations, especially in those under 6 months and between 1 and 3 years, as previously reported.^11^

Infections caused by PIV‐1 and PIV‐3 were mainly observed in infants under 6 months, whereas those caused by PIV‐2 were predominant not only in these patients but also in children aged 1 to 3 years. These results are comparable to those of Henrickson,^33^ who reported that children suffered more severe PIV‐1 and PIV‐2 infections in their first year, and PIV‐3 caused symptoms similar to those of RSV.

The overall rate of virus detection in the present study was higher in females than in males, in contrast with previously reported data.^4^, ^11^, ^19^, ^34^ This difference was observed only in adult female patients, which might be partly due to their having closer contact with young children compared to men.^27^

In our study, using antigen detection and viral isolation methods, co‐detections were obtained in 2.6% of positive samples. These data are similar to those obtained by other studies based on conventional methods^4^, ^10^ and lower than those only using molecular methods, where the frequency of co‐detections ranges from 10% to 50%.^14^, ^21^, ^22^ The clinical impact of co‐detection remains unknown, although a more severe clinical course has been associated with the detection of more than one respiratory virus.^35^

In this work, the most frequent co‐detection was of RSV and FLUAV, as reported in the literature,^5^ whereas other authors have found RV and AdV to be the most common,^21^, ^22^ attributing it to their year‐round circulation. In our study, cases of co‐detection were observed mainly during the winter months, the season associated with RSV and FLUAV circulation, and coinciding with the maximum number of virus detections.

The impact of the COVID‐19 pandemic, declared during the preparation of this manuscript, on society and the medical network changed the study of respiratory viral infections. Accordingly, the highest number of specimens for the detection of respiratory viruses received in our laboratory in 1 year during the previous 23‐year period was 4,883, whereas in 2020, it was 66,616. That year, the number of specimens processed for the detection of respiratory viruses other than SARS‐CoV‐2 was 3,808, and 862 such detections were made, 96.5% of them before the pandemic declaration (data not published). Little information is available about the circulation of respiratory viruses other than SARS‐CoV‐2 during the rest of 2020 because all efforts were directed to SARS‐CoV‐2.

A strength of this study is the use of gold standard methods, namely, viral culture and antigen detection immunofluorescence, together with the high number of samples and the long study period, as they provide a global vision of the prevalence of respiratory viruses in the area. A limitation of our study is that it was not based on systematic surveillance. Additionally, conventional methods are not the most suitable to detect certain viruses (e.g., rhinoviruses, coronaviruses, and some parainfluenza viruses), although they are more reliable than molecular methods for the detection of an infection in progress. The changes in methodology (mainly for FLUAV, FLUBV, or RSV) during the study period could have affected the number of detections, but the detection rates of the viruses in question were very similar in years when only conventional methods were used.

This long‐term study therefore broadens our knowledge of the prevalence of respiratory viruses in a Mediterranean region. The frequency of some viruses was constant throughout the study period, whereas others varied with the year. Clear demographic and seasonal patterns were evident for some viruses. During the influenza A pandemic, other respiratory viruses were detected in more than half of patients suffering respiratory infections. Other respiratory viruses were also detected during the first year of the SARS‐CoV‐2 pandemic.^36^ We conclude that patients suffering from severe respiratory infection should be screened for a wide range of respiratory viruses regardless of gender, age, and season.

## FUNDING INFORMATION

This work was supported by own funding.

### PEER REVIEW

The peer review history for this article is available at https://publons.com/publon/10.1111/irv.12972.

## Data Availability

Data sharing not applicable to this article as no datasets were generated or analysed during the current study
